# Evaluating the Value of Tissue-Based Assays in the Diagnosis of AE

**DOI:** 10.31083/RN48873

**Published:** 2026-07-21

**Authors:** Yaxiu Fang, Yan Tan, Guanghui Liu, Dong Zheng, Yuefeng Zhang, Xiaoming Zhou, Huarong Zhou, Pengbo Gao, Hui Zhang, Lin Chen, Yafang Hu, Yuping Ning

**Affiliations:** ^1^Department of Neurology, The Affiliated Brain Hospital of Guangzhou Medical University, 510370 Guangzhou, Guangdong, China; ^2^Department of Neurology, Nanfang Hospital, Southern Medical University, 510515 Guangzhou, Guangdong, China

**Keywords:** antibodies, autoimmune encephalitis (AE), cerebrospinal fluid, immunohistochemistry, tissue-based assay (TBA)

## Abstract

**Background:**

Tissue-based assay (TBA) results can reveal the existence of antibodies other than known antibodies. Clinically, some patients with negative cell-based assay (CBA) but positive TBA have similar symptoms to autoimmune encephalitis (AE), indicating that positive TBA results still provide a certain reference for clinicians to make decisions. Through a retrospective study, the diagnostic rate of AE in our center and the auxiliary diagnostic value of TBA for AE were analyzed. The sensitivity and specificity of cerebrospinal fluid TBA in the diagnosis of AE were also evaluated.

**Methods:**

Patients who met the diagnostic criteria for confirmed and possible AE in our center were retrospectively selected. The positive and negative rates of TBA in patients diagnosed with AE were analyzed, as were the positive rate and subcellular localization of TBA in possible AE patients with a negative CBA.

**Results:**

Among the 61 cases of confirmed AE, 48 cases had positive TBA, resulting in a detection rate of 79%. There were 13 cases (21%) with false negatives. Among the 115 possible AE patients with negative autoantibodies, 97 cases (45.8%) had positive TBA. Based on the cellular morphology revealed by staining, these cases were categorized into neuronal cell staining (48 cases, including 8 cases with membrane staining, 19 cases with nuclear staining, and 21 cases with cytoplasmic antibody), glial cell staining (15 cases), and simultaneous staining of neuronal and glial cells (4 cases); The area under the curve of AE confirmed by TBA positivity diagnosis was 0.672, 95% CI 0.594–0.749, *p* < 0.001, with sensitivity, specificity, and Youden’s index of 78.7%, 55.6%, and 0.343, respectively. In the logistic regression model for positive TBA and elevated cerebrospinal fluid leukocyte counts, the area under the curve (AUC) was 0.728, 95% CI 0.654–0.803, *p* < 0.001, with sensitivity and specificity, and Youden’s index of 57.4%, 79.5%, and 0.368, respectively.

**Conclusions:**

TBA serves as an effective complementary tool to CBA for AE diagnosis, with a sensitivity of 78.7% and specificity of 55.6%. Combining a positive TBA with an elevated cerebrospinal fluid (CSF) leukocyte count improved diagnostic specificity to 79.5%, suggesting that comprehensive evaluation integrating both assays can reduce missed diagnoses and optimize clinical management.

## 1. Introduction

Autoimmune encephalitis (AE) is a disorder that primarily affects the central nervous system (CNS). It is characterized by the production of autoantibodies targeting neuronal intracellular and cell-surface antigens, including neuronal surface antibodies (NSAbs). The clinical manifestations of AE can be diverse and include psychiatric and behavioral abnormalities, seizures, movement disorders, involuntary movements, cognitive impairments, memory deficits, speech disturbances, and alterations in consciousness, including coma [1,2]. The diagnostic hallmark of AE is the detection of immunoglobulin G (IgG) autoantibodies that specifically bind to intracellular or cell surface antigens on neurons [1].

Antibody detection plays a crucial role in clinical practice: it aids in establishing a clear diagnosis and differential diagnosis, and is significant for early disease detection, tumor screening, guiding diagnosis and treatment, and assessing prognosis. Currently, the most commonly used methods for antibody testing in clinical settings are the cell-based assay (CBA) and the tissue-based assay (TBA), both of which are indirect immunofluorescence techniques [3]. CBA focuses on specific antigen-antibody reactions. In this method, known antigens are overexpressed in 293 cells and then incubated with cerebrospinal fluid or blood samples from patients. If antibodies are present in the cerebrospinal fluid (CSF) or blood, fluorescence is produced. Its advantages include high sensitivity and specificity, but it is complex and time-consuming to perform. On the other hand, TBA uses tissue slices from animals (such as mouse hippocampus or cerebellum) as antigen substrates on glass slides. After a series of treatments, the slices are incubated with a patient’s CSF or serum. If antibodies are present, fluorescence is produced.

CBA can detect only about 30 known antibodies, leaving many unknown antibodies undiscovered. Recent advances in neural antibody testing have emphasized the importance of comprehensive testing strategies, with recommendations suggesting that TBA should be used alongside CBA to maximize diagnostic sensitivity and specificity [4,5]. Therefore, when CBA is negative, but the patient’s clinical manifestations meet the criteria for autoimmune encephalitis, TBA results should be considered. Because a positive TBA indicates the presence of antigen-antibody reactions but does not specify the type of antibody (it could be a known or unknown antibody), it can provide information about the different brain regions and subcellular localization of the antibodies. TBA results play an important role in disease warning, guiding diagnosis and treatment, and evaluating prognosis. Previous studies have demonstrated that TBA is a valuable screening tool for AE diagnosis and can broaden the search for neurological autoimmunity, although paired serum and CSF testing is recommended to maximize diagnostic accuracy [6]. It is also worth noting that AE cannot be ruled out based solely on a negative antibody test result.

We conducted a retrospective study to explore the diagnostic rate of AE in our center and the auxiliary diagnostic value of TBA for AE. This is particularly relevant given recent studies highlighting the clinical importance of TBA in identifying patients who may benefit from immunotherapy even in the absence of known antibodies [7,8]. The proportion of known antibody-negative but positive TBA patients, and the subcellular localization of the target antigen in positive TBA patients, were analyzed.

## 2. Methods

### 2.1 Patients and CSF

This study was a single-center retrospective cohort study. We retrospectively selected patients from the Department of Neurology or Psychiatry at The Affiliated Brain Hospital of Guangzhou Medical University. The inclusion criteria were based on the Chinese Expert Consensus for Diagnosis and Treatment of Autoimmune Encephalitis in 2017 and 2022, including patients who met the diagnostic criteria for definite AE or possible AE. This study was conducted and is reported in accordance with the STROBE statement for cohort studies (**Supplementary Material-STROBE_Checklist**).

### 2.2 Pattern-Specific Primary Antibody Screening Using an Indirect Immunofluorescence Assay in Mouse Brain Tissue

 C57BL/6J mice (male, age: 8–10 weeks, weight: 20–25 g, Beijing, China) were anesthetized with sodium pentobarbital (50 mg/kg body weight, i.p. Solarbio, Beijing, China). After confirmation of adequate anesthesia depth by the absence of the pedal reflex, mice were euthanized by an overdose of sodium pentobarbital (150 mg/kg body weight, i.p.) and then transcardially perfused with ice-cold 0.9% saline (20 mL). The brains were rapidly dissected, embedded in optimal cutting temperature (OCT) compound(4583, Sakura Finetek, Torrance, CA, USA), and snap-frozen using isopentane pre-cooled in liquid nitrogen (−80 °C). Frozen brain sections were cut at 5 μm using a cryostat (Leica CM1950, Leica Biosystems, Wetzlar, Germany) maintained at –20 °C, and were then mounted on poly-L-lysine-coated glass slides (P8920, Sigma-Aldrich, St. Louis, MO, USA). Sections were stored at –80 °C until use. For each test, 50 μL of CSF at a dilution of 1:1 was incubated with mouse brain sections at 37 °C for 1 h. After washing three times with saline, each section was further incubated with 50 μL of diluted DyLight 488-labeled goat anti-human IgG secondary antibody (1:200 dilution, ab97003, Abcam, Cambridge, MA, USA) at 37 °C for another 1 h. Then, the sections were washed three times again and photographed under a DM3000 fluorescence microscope (Leica Biosystems).

### 2.3 CBA

CBA is the standard method for the diagnosis of known autoantigens in patients’ samples (serum or CSF), in which HEK293 cells were used, and each known autoantigen is overexpressed separately. CSF samples from patients were tested for known autoantibodies using CBA, including anti-N-methyl-D-aspartate receptor (NMDAR), leucine-rich glioma inactivated 1 (LGI1), contactin-associated protein-like 2 (Caspr2), gamma-aminobutyric acid receptor (GABAR), α-amino-3-hydroxy-5-methyl-4-isoxazolepropionic acid receptor (AMPAR), metabotropic glutamate receptor 5 (mGluR5), GAD65, glutamic acid decarboxylase 65 (GAD65), aquaporin-4 (AQP4), and myelin oligodendrocyte glycoprotein (MOG) antibodies, using Euroimmun assay kits (Autoimmune Encephalitis Mosaic 1, FA 1121-1005-1, Euroimmun AG, Lübeck, Germany) or as previously reported. Although AQP4 and MOG antibodies are primarily diagnostic biomarkers for neuromyelitis optica spectrum disorder (NMOSD) and myelin oligodendrocyte glycoprotein antibody-associated disease (MOGAD), respectively, with serum testing recommended as the gold standard due to higher sensitivity, these antibodies were included in our CSF-based panel for comprehensive differential diagnosis of CNS autoimmune disorders that may present with encephalitic features. We acknowledge that CSF testing alone has limited sensitivity for these antibodies, and positive CSF results should ideally be confirmed with paired serum testing in clinical practice.

### 2.4 Statistical Methods

SPSS 25.0 (IBM Corp., Armonk, NY, USA) and GraphPad Prism 8.0.2 (GraphPad, La Jolla, CA, USA) were used for data analysis and figure plotting. A two-tailed *p* ≤ 0.05 was considered statistically significant. MedCalc 20.1.0 (MedCalc, Mariakerke, Belgium) was used to plot the receiver operating characteristic (ROC) curve.

The normality of the distribution of continuous variables was assessed using the Kolmogorov-Smirnov test. Normally distributed numerical variables were presented as mean ± standard deviation and analyzed using independent-sample *t*-tests for intergroup comparisons. Non-normally distributed numerical variables were presented as median (interquartile range) and analyzed using non-parametric Mann-Whitney U tests for intergroup comparisons. Categorical variables were presented as frequency counts (percentages) and analyzed using the chi-square test (Fisher’s exact test when expected frequency <5) for intergroup comparisons. Biological replicates were used in this study, with each sample obtained from an independent subject, and no technical replicates were performed.

## 3. Results

### 3.1 Demographic Data and Research Process

**Fig. 1.**
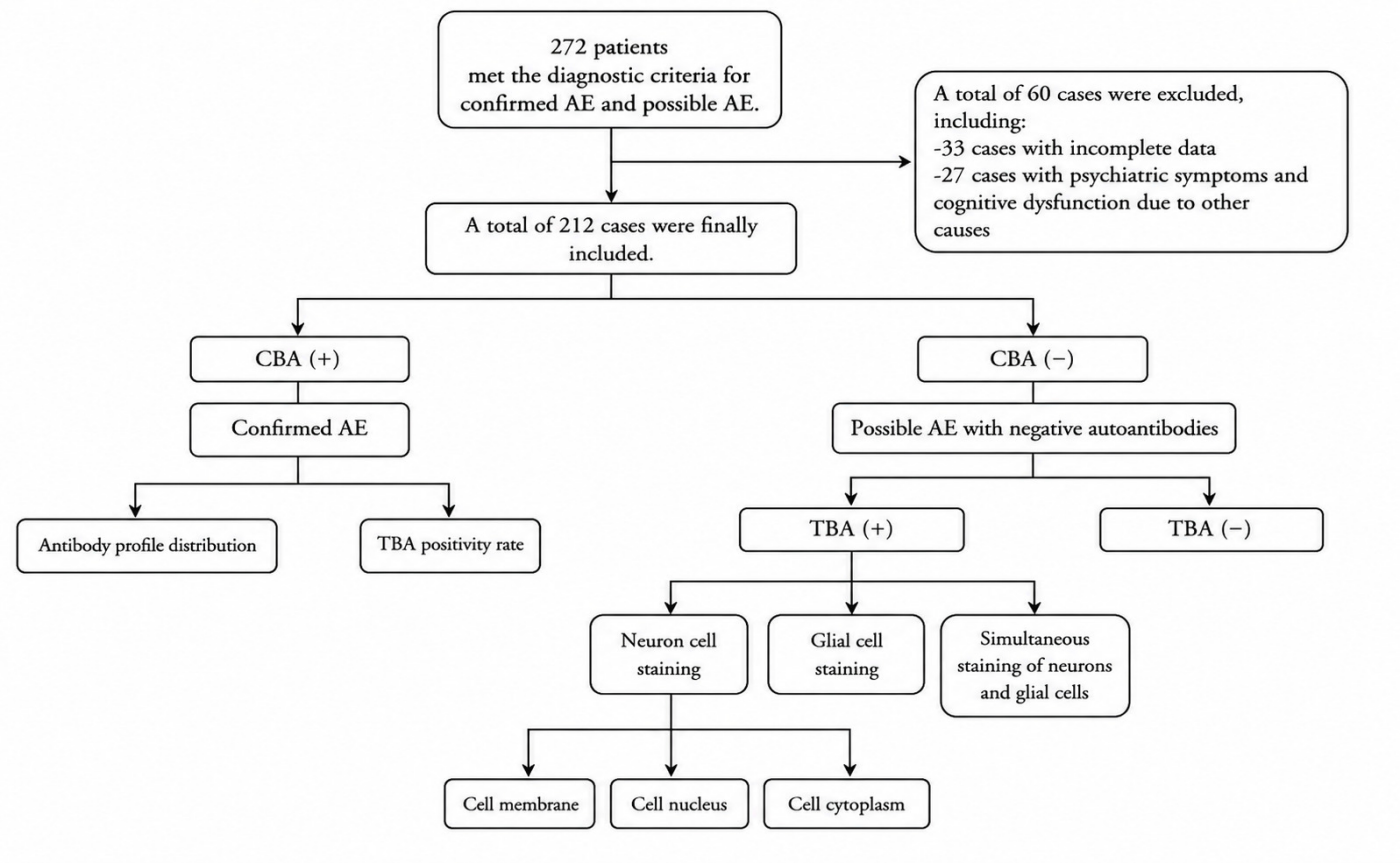
**Flow chart of study patient inclusion and exclusion**. AE, autoimmune encephalitis; CBA, cell-based assay; TBA, tissue-based assay; +, positive; –, negative.

A total of 212 patients were included in this study, with a mean age of 32 (21.5~52) years. Among them, 116 (54.7%) were male. The inclusion, exclusion, and research process are illustrated in Fig. 1, and the demographic data and baseline clinical characteristics are presented in Table 1.

**Table 1.** **Demographic data and clinical baseline data**.

Variable		Values (%)
Age (years)		32 (21.5~52)
Sex,* n (%)*	Female	96 (45.3)
	Male	116 (54.7)
Clinical symptoms, *n (%)*	Fever	32 (15.1)
	Headache	14 (6.6)
	Psychiatric abnormalities	198 (93.4)
	Seizures	46 (21.7)
	Memory impairment	49 (23.1)
	Sleep disorders	126 (59.4)
Cerebrospinal fluid findings, *n (%)*	Elevated white blood cell count	90 (42.5)
	Increased protein level	79 (37.3)
Neuroimaging findings, *n (%)*	Inflammatory changes	46 (28.8)
	Cerebral white matter lesions	49 (23.1)
	Brain atrophy	46 (21.7)
Confirmed AE, *n (%)*		61 (29.0)

All patients had CSF collected for TBA and CBA testing. Among them, 61 cases (29%, 61/212) were diagnosed as definite AE based on positive CBA results. Additionally, 151 cases were classified as possible AE with negative CBA results. Subsequently, we analyzed the distribution of autoantibody patterns and the positivity rate of TBA in the 61 confirmed AE cases. We also conducted a retrospective analysis of the final diagnosis in 67 TBA positive patients among the suspected AE cases with negative autoantibodies. Furthermore, we examined the characteristics and treatment outcomes of cases with TBA-positive staining in neuronal cell membranes, cytoplasm, and nuclei.

### 3.2 Positivity Rate and Distribution of Autoantibody Patterns in Confirmed AE

We analyzed the distribution of autoantibody patterns in the 61 confirmed AE cases. NMDAR-IgG was the most common antibody, present in 50 individuals (81.97%, 50/61). NMDAR+MOG was found in 3 individuals (4.92%); 2 individuals MOG positive (3.28%); 1 individual was collapsin response mediator protein 5 (CRMP5) positive (1.64%); 1 individual LGI1 positive (1.64%); 1 individual was GAD65 positive (1.64%); 1 individual was GABAbR positive (1.64%); 1 individual was GABAARγ2 positive (1.64%); and 1 individual was positive for both CRMP5 and Zic family zinc finger proteins (Zic) antibodies (1.64%). The distribution is illustrated in Table 2.

**Table 2.** **Statistical chart of known antibody detection in diagnosed AE patients**.

Autoimmune encephalitis	Antibody	*n*
Definite AE (*n = 61*)		
	NMDAR-IgG	50
	NMDAR-IgG+MOG-IgG	3
	MOG-IgG	2
	CRMP5-IgG	1
	LGI1-IgG	1
	CV2-IgG+Zic-IgG	1
	GABAbR-IgG	1
	GABAARγ2-IgG	1
	GAD65-IgG	1

### 3.3 Detection Rate of TBA in Confirmed AE

Among the 61 cases of confirmed AE, 48 had a positive TBA, resulting in a detection rate of 79%. There were 13 cases (21%) with false negatives, as shown in **Supplementary Table 1**. This indicated that CBA had higher specificity and sensitivity than did TBA. This discordance likely reflected technical limitations of TBA rather than false-positive CBA results. Potential reasons for TBA false negativity included lower analytical sensitivity for low-titer antibodies than CBA, poor preservation or low expression of some antigens in fixed tissue sections, reduced antibody binding affinity to native tissue antigens versus overexpressed CBA antigens, technical factors including tissue quality and detection thresholds, and difficulty detecting antibodies targeting specific subcellular compartments. All 13 negative TBA cases had clinical presentations and outcomes consistent with definite AE, supporting the validity of CBA results.

### 3.4 TBA Positivity Rate and Subcellular Localization in Possible AE Patients With Negative Autoantibodies

In this study, among the 151 possible AE patients with negative autoantibodies, 67 cases (44.37%) had positive TBAs, as shown in **Supplementary Table 2**. Based on the cellular morphology of staining, these cases were categorized into neuronal cell staining (48 cases, including 8 cases with membrane staining, 19 cases with nuclear staining, and 21 cases with cytoplasmic antibody), glial cell staining (15 cases), and simultaneous staining of neuronal and glial cells (4 cases). The percentages for each group are shown in **Supplementary Table 3** and **Supplementary Table 4**.

Additionally, we conducted a retrospective analysis of the clinical characteristics, final diagnosis, and treatment outcomes of the 67 patients with positive TBA among the possible AE patients with negative autoantibodies. In combination with the cellular and subcellular localization of TBA staining, we found the potential existence of missed diagnoses by CBA or the possibility of unknown antibodies.

Table 3 presents the clinical data of eight suspected AE patients with negative known antibodies but positive TBA staining in neuronal cell membranes. The discharge diagnoses for these cases include viral encephalitis (2 cases), neurosyphilis (1 case), epilepsy (3 cases) and delirium (2 cases).

**Table 3.** **TBA showed positive cell membrane antibodies in suspected AE**.

Patient	Sex	Age (year)	Main symptoms	Diagnosis	CSF (WBC)	MRI	Immunotherapy	Prognosis (mRS)
P80	F	15	Acute-onset behavioral abnormalities with delayed reactions lasting 1+ week	Viral encephalitis	10 × 10^6^/L	Suspected increased FLAIR signal in bilateral thalamus, inflammation?	Y	0
P62	F	51	Fever, disordered speech and behavior for 20+ days	Viral encephalitis	5 × 10^6^/L	1. Mild small ischemic lesions in the left parietal lobe; 2. Mild brain atrophy	N	1
P97	M	48	Memory decline with behavioral abnormalities for 2 months	Neurosyphilis	7 × 10^6^/L	Multiple abnormal signals in the brain with suspected linear enhancement of some intracranial soft meninges and dura	N	1
P58	F	32	Acute-onset paroxysmal behavioral abnormalities for 1 week	1. Symptomatic epilepsy (psychomotor seizures); 2. Systemic lupus erythematosus	3 × 10^6^/L	Abnormal density shadow in the left parietal lobe white matter, suggestive of demyelination	Y	0
P59	F	20	Recurrent seizures for 11 years, refusal to eat, aphasia for 10+ days	1. Symptomatic epilepsy; 2. Organic brain disorder	2 × 10^6^/L	No significant abnormalities observed	N	2
P60	M	26	Recurrent limb seizures for 10 years, acute-onset behavioral abnormalities for 4 days	1. Symptomatic epilepsy; 2. Organic brain disorder	5 × 10^6^/L	No significant abnormalities observed	N	2
P66	F	46	Recurrent fever and cough for 3 weeks, incoherent speech for more than 1 day	1. Delirium; 2. Respiratory tract infection	1 × 10^6^/L	Small spot lesions in bilateral frontal lobe white matter, suggestive of demyelination	N	0
P182	F	64	Acute-onset behavioral abnormalities for 3 days	1. Delirium; 2. Respiratory tract infection	2 × 10^6^/L	Mild widening of bilateral temporal sulci	N	1

F, female; M, male; FLAIR, fluid-attenuated inversion recovery; Y, yes; N, no.

### 3.5 Clinical Manifestations and Prognosis of Patients Positive for Cytoplasmic Antibody as Indicated by TBA

**Supplementary Table 5** presents the clinical data of 21 patients with suspected AE who tested positive for cytoplasmic antibody by TBA but negative for CBA. Among them, further specific diagnoses were made in 8 cases, including neurosyphilis (6 cases), herpes simplex virus encephalitis (1 case), and psychiatric disorder due to thyroid dysfunction (1 case). The remaining 13 cases were discharged without a definitive etiology and were diagnosed as idiopathic encephalitis (3 cases), epilepsy (6 cases), and delirium (4 cases).

### 3.6 Clinical Manifestations and Prognosis of Patients With TBA Suggestive of Nuclear Antibody-Positive Cells

**Supplementary Table 6** shows the clinical data of 19 patients with suspected AE who had positive TBA staining in the nuclei of neuronal cells with negative CBA. Among them, 11 cases could be further clearly diagnosed, which were neurosyphilis (5 cases), herpes zoster virus encephalitis (1 case), rickettsial infection (1 case), tuberculous meningitis (1 case), septic meningitis (1 case), venous sinus thrombosis (1 case), and psychiatric disorders due to SLE (1 case). There were also 8 cases of unspecified etiology, which were epilepsy of unknown cause (2 cases) and encephalitis of unknown cause (6 cases).

### 3.7 Value of TBA Positivity for AE Diagnosis

Although the diagnostic value and specificity of CBA in AE are now well recognized, there are still reports indicating that the diagnostic rate of CBA for AE is only about 20%, which may be an underestimate. Therefore, we need to have tests with higher diagnostic rates, and we need to know how sensitive and specific TBA is in the diagnosis of AE. Therefore, in this study, we further analyzed the diagnostic value of TBA in the diagnosis of AE by using TBA as an overall indicator in the included 212 patients with possible AE.

First, we divided the included 212 patients who met the diagnostic criteria for possible AE into positive TBA and negative TBA groups, with 115 (54.2%) positive TBA and 97 (45.8%) negative TBA patients. We found that TBA-positive patients were significantly more likely to present with mood abnormalities and significantly less likely to have fever than TBA-negative patients. Among all included patients with possible AE, positive TBA patients had significantly higher CSF leukocyte counts and total protein levels than did negative TBA patients, and more positive patients were ultimately diagnosed with AE than were TBA-negative patients (Table 4).

**Table 4.** **Clinical data of the TBA positivity group and the TBA negativity group**.

Variable		TBA positivity (n = 115)	TBA negativity (n = 97)
Age, years		33 (22~54.25)	31 (20.5~47.5)
Sex, Cases (%)	Female	51 (44.3)	44 (45.4)
	Male	64 (55.7)	53 (54.6)
Clinical presentation, Cases (%)	Fever	8 (7.0)	24 (24.7)
	Headache	8 (7.0)	6 (6.2)
	Psychiatric Abnormalities	92 (80.0)	80 (82.5)
	Epilepsy	25 (21.7)	21 (21.6)
	Memory Decline	25 (21.7)	14 (14.4)
	Cognitive Impairment	8 (7.0)	2 (2.1)
	Mood Disturbances	21 (18.3)	5 (5.2)
	Sleep Disorders	5 (4.3)	6 (6.2)
CSF routine, Cases (%)	Elevated WBC Count	66 (57.4)	24 (24.7)
	Elevated CSF total protein	54 (47.0)	25 (25.8)
	Oligoclonal Bands	1 (0.9)	0 (0.0)
Neuroimaging findings, Cases (%)	Inflammatory Changes	28 (24.3)	18 (18.6)
	White Matter Lesions	30 (26.1)	19 (19.6)
	Brain Atrophy	29 (25.2)	17 (17.5)
Confirmed AE, Cases (%)		48 (41.7)	13 (13.4)

CSF, cerebrospinal fluid; WBC, white blood cells.

**Fig. 2.**
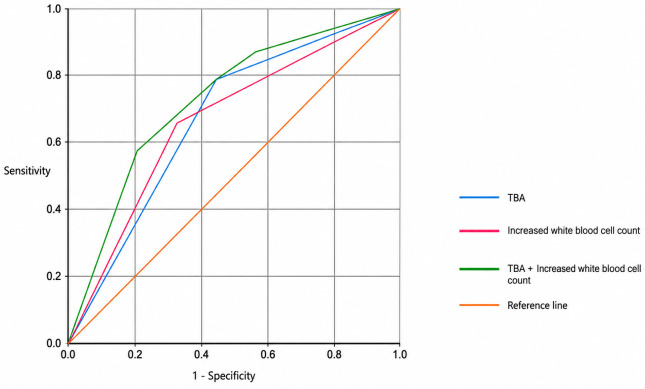
**ROC curve analysis of TBA positivity and CSF white blood cell count was performed based on a stepwise logistic regression model for the diagnosis of AE. **ROC, receiver operating characteristic.

According to the baseline information of the two groups, variables with a *p* < 0.05 (increased WBC count and TBA positivity) were included in the multivariate logistic stepwise regression analysis model along with positive TBA. The results were that positive TBA (adjusted odds ratio (aOR) 3.423, 95% CI 1.660–7.058, *p* = 0.001) and elevated CSF leukocyte count (aOR 2.852, 95% CI 1.469–5.536, *p* = 0.002) were both predictors of confirmed AE (Table 5). The ROC curves for positive TBA and elevated CSF leukocyte count for diagnosis of confirmed AE were further performed (Fig. 2). The area under the curve of AE confirmed by positive-TBA diagnosis was 0.672, 95% CI 0.594–0.749, *p* < 0.001, and sensitivity, specificity, and Youden’s index were 78.7%, 55.6%, and 0.343, respectively. Elevated cerebrospinal fluid (CSF) leukocyte counts were more effective in diagnosing and confirming AE, with an area under the curve of only 0.666, 0.584–0.747, *p* < 0.001, and sensitivity, specificity, and Youden’s index were 65.6%, 67.5%, and 0.331, respectively. In the logistic regression model for positive TBA and elevated CSF leukocyte counts, the area under the curve (AUC) was 0.728 (0.654–0.803, *p* < 0.001), with sensitivity and specificity of 57.4%, 79.5%, and Youden’s index of 0.368, respectively (Table 6).

**Table 5.** **Univariate and multivariate logistic regression of confirmed AE**.

Variant	Univariate logistic regression		Multivariate logistic regression	
	OR (95% CI)	*p*	aOR (95% CI)	*p*-value
Fever	1.046 (0.372–2.937)	0.932		
Emotional abnormality	1.349 (0.520–3.501)	0.538		
Cognitive impairment	1.377 (0.321–5.915)	0.667		
Increased WBC count	3.511 (1.675–7.357)	0.001	2.852 (1.469–5.536)	0.002
Elevated CSF total protein	0.576 (0.275–1.209)	0.145		
TBA positivity	3.400 (1.590–7.271)	0.002	3.423 (1.660–7.058)	0.001

Note: multivariate logistic regressions were modeled using stepwise forward regression.

**Table 6.** **The diagnostic value of TBA positivity and CSF white blood cell count increase stepwise logistic regression model (positive TBA and CSF white blood cell count increase) for the diagnosis of AE**.

Variant	AUC (95% CI)	Sensitivity (%)	Specificity (%)	Youden’s index
TBA positivity	0.672 (0.594–0.749)	78.7	55.6	0.343
Elevated white blood cell count	0.666 (0.584–0.747)	65.6	67.5	0.331
TBA+elevated white blood cell count	0.728 (0.654–0.803)	57.4	79.5	0.368

AUC, area under the curve.

## 4. Discussion

Currently, CBA and TBA are both indirect immunofluorescence techniques. CBA focuses on specific antigen-antibody reactions. TBA uses tissue slices from animals (such as mouse hippocampus or cerebellum) as antigens. Positive TBA indicates the presence of antigen-antibody reactions but does not specify the type of antibody (it could be a known or unknown antibody). It plays an important role in disease warning, guiding diagnosis and treatment, and evaluating prognosis. And it is also worth noting that AE cannot be ruled out based solely on a negative antibody test result. As TBA enables screening for unknown autoantibodies, we suggest a stepwise combination of TBA with commercial CBA as a standard diagnostic approach [9].

CBA or immunoblot testing covers nearly 20 known autoantibodies, achieving a total detection rate of less than 20% in early diagnosis when combined with clinical features of AE. Negative CBA results may include unidentified antibodies. Additionally, there may be differences in the sensitivity and specificity of different commercial test kits, which could lead to false-negative results. For example, studies have reported a 12% missed detection rate in positive cases using serum testing [10]. Two research teams conducted antibody testing using commercial CBA kits on patients with clinical syndromes consistent with anti-LGI1 encephalitis, with sensitivities of 53% and 57%, respectively, indicating the existence of missed detections [11]. Immunohistochemistry on mouse brain slices may have improved the diagnostic accuracy of neuronal antibodies in AE patients and served as a valuable supplementary method [12].

Our study included 212 patients who met the possible AE diagnostic criteria, including 116 males, with an average age of 32 (21.5~52) years. Among them, 61 cases (29%) were diagnosed as definite AE based on positive CBA results, and the remaining 151 cases were identified as possible AE patients with negative CBA results for autoantibodies. Among the 151 possible AE patients with negative CBA, 67 cases (44.37%) showed a positive TBA. Based on the staining pattern, positive TBA was divided into 48 cases of neuronal cell membrane staining (including 8 cases of cell membrane staining, 19 cases of nuclear staining, and 21 cases of cytoplasmic antibody), 15 cases of glial cell staining, and 4 cases of staining in both neuronal and glial cells. A retrospective analysis of 8 patients with negative CBA but positive TBA staining on neuronal cell membranes indicated that only 2 received immunotherapy and had a good prognosis. Among the other 6 who did not receive immunotherapy, 2 (1 case of viral encephalitis and 1 case of delirium secondary to respiratory tract infection, treated with antiviral and antibacterial therapy) had a good prognosis, and the remaining 3 had poor prognoses with varying degrees of cognitive impairment and social dysfunction. This suggested the possibility of missed diagnoses or unknown antibodies with CBA, leading to delayed initiation of immunotherapy and affecting treatment efficacy. Additionally, a retrospective clinical analysis was conducted on patients with positive TBA staining in neuronal cytoplasm and nuclei, showing clinical significance in suspected AE patients with known antibody negativity. The prognosis of the 67 cases of known negative antibody but positive TBA requires further follow-up observation.

Although the pathological mechanisms of the effect of antibodies against neuronal cell membrane, intracellular or nuclear antigens, and glial cells detected in CSF are not yet clear, antibodies targeting cell surface or axonal antigens are known to interfere with neuronal function and have pathogenic effects. The pathogenicity of antibodies against intracellular neuronal antigens has not been determined. Therefore, when TBA staining is positive on neuronal cell surfaces, the finding can be considered to involve pathogenic potential. Thus, TBA is a valuable screening tool and auxiliary diagnostic method for suspected AE patients, providing a basis for the potential discovery of new antibodies. We should emphasize the importance of TBA testing, especially for the small number of cases that have positive TBA but negative CBA. Additional targeted CBA testing can be performed in these cases, taking into account the patient’s clinical symptoms, along with electroencephalography and cranial imaging to aid in the discovery of new antigens and to obtain a definitive diagnosis.

It should be noted that the interpretation of CBA and TBA results needs to be combined with clinical information. For example, a single positive result does not necessarily mean an AE diagnosis. For instance, if a patient is positive for NMDAR antibodies in serum but is negative in CSF, and does not exhibit neurological or psychiatric symptoms, anti-NMDAR encephalitis cannot be diagnosed [13]. Some patients may continue to have detectable antibodies in blood or CSF for a certain period even after recovery, although the exact duration is still unknown. Furthermore, the ideal testing method for CBA and TBA is paired testing using both blood and CSF samples since many known AE antibodies are synthesized extrathecally and appear earlier and at higher titers in blood, making them easier to detect. A major limitation of this study is that only retained CSF samples were included, and no blood samples were preserved. In the future, it is necessary to further analyze the value of positive detection in blood and CSF for possible AE diagnosis in order to understand the sensitivity and specificity of serum TBA in AE diagnosis. That requires larger studies with paired blood and CSF samples. If CSF neuroreactivity is detected, initiation of immunotherapy may be considered even in cases of unidentiﬁed antigen speciﬁcity-consistent with the concept of seronegative autoimmune encephalitis and in accordance with approaches taken prior to the discovery of NMDAR encephalitis [12,14].

This study was retrospective, so most of the suspected AE patients with positive TBA and negative CBA results did not receive immunotherapy with corticosteroids or intravenous immunoglobulins. Therefore, whether immunotherapy could have improved the prognosis of such patients remains unknown. We could not determine at present if TBA positivity should be used as a reference for deciding whether to initiate immunotherapy, and further prospective studies are needed to observe this. Recent best practice recommendations have emphasized that neural antibody testing should incorporate tissue-based assays along with cell-based assays that include a broad range of antigens [4,15].

Although the value and specificity of CBA in AE diagnosis have been recognized, reports have suggested that the diagnostic rate of CBA for AE is only around 20%, indicating the possibility of missed diagnoses. A large-scale evaluation (1949 patients) demonstrated that 67% of individuals who were indirect immunofluorescence assay (IFA) positive but CBA negative exhibited clinically relevant autoimmunity, with 92% responding well to immunotherapy [7]. Similarly, recent studies from Asian cohorts have reported comparable findings regarding antibody detection rates and clinical outcomes [16,17]. A retrospective evaluation of 159 patients with suspected AE confirmed the role of TBA as a screening tool, noting that testing serum showed higher sensitivity than did testing CSF, although CSF had higher specificity [6].

What is the diagnostic value of TBA in AE? Currently, there is a lack of large-scale studies on the sensitivity and specificity of TBA in AE diagnosis, so we conducted a comparative analysis of the clinical characteristics between 212 possible AE patients having TBA positive and negative results: (a) among the possible AE patients, those with positive TBA results had a higher proportion of emotional abnormalities than did the negative TBA group, whereas fever was less common; (b) positive TBA patients had significantly higher CSF white blood cell counts and total protein levels than did the negative TBA group. In addition, (c) we performed a systematic evaluation of the sensitivity and specificity of TBA in diagnosing AE among the 212 possible AE patients, and found that a positive TBA was a predictive factor for definite AE, with a sensitivity of 78.7% and specificity of 55.6%. These findings were consistent with recent reports that emphasized that seronegative AE represents a substantial proportion of cases and that comprehensive antibody testing, including TBA, is essential for accurate diagnosis [18]. If positive TBA was combined with an elevated CSF white blood cell count, the specificity could be increased to 79.5%.

Based on our research findings, TBA can serve as a valuable screening tool for suspected AE patients and can assist in improving the diagnostic rate of AE when used in conjunction with CBA, thus avoiding missed diagnoses and aiding clinicians in making diagnostic and treatment decisions. Recent advances in autoimmune encephalitis research have led to the discovery of numerous novel autoantibodies and improved understanding of disease mechanisms [8]. The field has also seen important developments in standardization of antibody testing methodologies [19], which are critical for ensuring diagnostic accuracy across different laboratories. It should be noted that although our antibody panel included AQP4 and MOG, which are primarily associated with NMOSD and MOG-associated disease, respectively, their inclusion allowed for comprehensive differential diagnosis of CNS inflammatory conditions that may present with encephalitic features. The 21% false-negative rate of TBA in CBA-confirmed cases suggested that TBA should be used as a complementary screening tool rather than a replacement for CBA. The higher analytical sensitivity and specificity of CBA for known antibodies make it the gold standard for antibody confirmation, whereas TBA provides broader screening capability for unknown antibodies.

Several limitations of this study should be acknowledged. (1) Our study used CSF samples alone without paired serum testing. It is well established that the ideal testing method for both CBA and TBA involves paired analysis of blood and CSF, as this approach significantly improves diagnostic sensitivity. Many known AE-associated antibodies are synthesized extrathecally and may appear earlier or at higher titers in the serum, making them easier to detect. Due to the retrospective nature of this study, we relied on archived CSF samples, as paired serum samples were not systematically preserved. Future prospective studies should incorporate paired serum and CSF analysis to fully evaluate the diagnostic synergy of TBA across both sample types and to better define the sensitivity and specificity of serum TBA in the diagnosis of AE. (2) Our CBA panel, although comprehensive for commonly recognized AE antibodies, did not include all recently described rare neuronal antibodies. Some TBA-positive/CBA-negative cases may harbor antibodies not included in our testing panel, highlighting the potential value of TBA in identifying patients who may benefit from extended antibody characterization or novel antibody discovery. Future studies incorporating broader antibody panels or proteomics-based approaches may help elucidate the antigenic targets in these cases. (3) It is important to acknowledge that non-specific background staining is a recognized limitation of TBA that can complicate result interpretation. In this study, several measures were implemented to minimize false-positive results and distinguish true autoantibody binding from non-specific staining. All TBA interpretations were performed by two experienced technicians blinded to clinical information, with discrepancies resolved by a senior reviewer. Standardized protocols, including optimized CSF dilution, consistent incubation conditions, and appropriate controls, were employed. True positive results were identified by characteristic staining patterns with distinct cellular localization (membrane, cytoplasmic, nuclear, or glial), clear cellular morphology, and reproducible regional distribution, whereas non-specific binding typically presents as diffuse background fluorescence without recognizable cellular architecture. The false-positive rate of TBA in clinical practice remains a concern, as reflected by the 55.6% specificity observed in our study. This suggested that approximately 44% of TBA-positive results in CBA-negative patients may have represented non-specific binding rather than true unknown autoantibodies. Combining TBA results with other CSF inflammatory markers, particularly elevated white blood cell count, substantially improved specificity to 79.5%. These findings suggested that TBA should be interpreted within the broader clinical and laboratory context, and positive results should prompt careful correlation with clinical presentation, neuroimaging, and other supportive findings before informing treatment decisions. (4) Our CBA panel included AQP4 and MOG antibodies, which were tested in CSF samples only. We acknowledge that current diagnostic guidelines recommend serum-based testing as the gold standard for these antibodies due to significantly higher sensitivity than CSF testing. The inclusion of these antibodies in our panel was intended for comprehensive differential diagnosis rather than primary diagnosis of NMOSD or MOGAD. Future studies should incorporate paired serum and CSF testing for these antibodies to ensure optimal diagnostic accuracy.

## 5. Conclusions

CBA testing alone confirmed AE in only 29% of suspected cases, whereas TBA detected positive results in 44.37% of CBA-negative possible AE patients. CSF-TBA demonstrated 78.7% sensitivity and 55.6% specificity for AE diagnosis, with specificity increasing to 79.5% when combined with an elevated CSF white blood cell count. These findings support the idea that TBA is a valuable complementary screening tool to CBA, particularly for identifying patients with potential unknown antibodies, thereby reducing missed diagnoses and guiding clinical decision-making in autoimmune encephalitis.

## Data Availability

The datasets generated and analysed during the current study are not publicly available because they contain clinical information that could compromise patient privacy and confidentiality, but they are available from the corresponding authors on reasonable request.

## Abbreviations

AE, autoimmune encephalitis; CNS, central nervous system; NSAbs, neuronal surface antibodies; IgG, immunoglobulin G; CBA, cell-based assay; TBA, tissue-based assay; NMDAR, anti-N-methyl-D-aspartate receptor; LGI1, leucine-rich glioma inactivated 1; Caspr2, contactin-associated protein-like 2; GABAR, gamma-aminobutyric acid receptor; AMPAR, α-amino-3-hydroxy-5-methyl-4-isoxazolepropionic acid receptor; mGluR5, metabotropic glutamate receptor 5; GAD65, glutamic acid decarboxylase 65; MOG, myelin oligodendrocyte glycoprotein; AQP4, aquaporin-4; ROC, receiver operating characteristic; CSF, cerebrospinal fluid; WBC, white blood cells.
