# Supplementary material for: Evaluating the Value of Tissue-Based Assays in the Diagnosis of AE
Source: Rev Neurol. 2026 Jul 21;81(7):48873. doi: 10.31083/RN48873 (PMC13421102; doi:10.31083/RN48873)
Supplement: Supplementary file 1 [file 1576-6578-81-7-48873-s1.zip › Supplementary Material.docx]

**Evaluating the Value of Tissue-Based Assays in the Diagnosis of AE**

[Supplementary Table 1 The positive rate of TBA in diagnosed AE patients 2](#_Toc229664927)

[Supplementary Table 2 The positive rate of TBA in possible AE patients 2](#_Toc229664928)

[Supplementary Table 3 Colored Cell Localization in Patients with possible AE patient 2](#_Toc229664929)

[Supplementary Table 4 Subcellular localization of TBA positivity neurons 2](#_Toc229664930)

[Supplementary Table 5 TBA showed positive cytoplasmic antibodies in patients with suspected AE 3](#_Toc229664931)

[Supplementary Table 6 TBA showed a suspected AE patient with positive nuclear antibodies 7](#_Toc229664932)

**Supplementary Table 1** The positive rate of TBA in diagnosed AE patients

| **Autoimmune encephalitis** | **TBA (+)** | **TBA (-)** |
| --- | --- | --- |
| Definite AE (n=61) | 48 (79%) | 13 (21%) |
| Autoantibody-negative but probable AE (n=151) | 67 (44%） | 84 (56%) |

**Supplementary Table 2** The positive rate of TBA in possible AE patients

| **Possible AE (CBA negativity)** | **TBA (+)** | **TBA (-)** |
| --- | --- | --- |
| n=151 | 67 (44%) | 84 (56%) |

**Supplementary Table 3** Colored Cell Localization in Patients with possible AE patient

| **Possible AE (n=151)** | **Colored cell Localization** | **N** |
| --- | --- | --- |
| Autoantibody-negative but TBA positivity (n=67) | Neuron cell | 48 (72%) |
|  | Glial cell | 15 (22%) |
|  | Neurons and glial cells | 4 (6%) |

**Supplementary Table 4** Subcellular localization of TBA positivity neurons

| **POSSIBLE AE（n=151）** | **Subcellular localization** | **N** |
| --- | --- | --- |
| Autoantibody-negative but TBA positivity (n=48) | Cell membrane | 8 (17%) |
|  | Cell cytoplasm | 21（44%） |
|  | Cell nucleus | 19（39%） |

**Supplementary Table 5** TBA showed positive cytoplasmic antibodies in patients with suspected AE

| **Patient** | **Sex** | **Age (y)** | **Main Symptoms** | **Diagnosis** | **CSF (WBC)** | **MRI** | **Immunotherapy** | **Prognosis (mRS)** |
| --- | --- | --- | --- | --- | --- | --- | --- | --- |
| P56 | F | 28 | Recurrent loss of consciousness for 2 years, recurrent episodes for 1 week | Symptomatic epilepsy | 2×10^6/L | No significant abnormalities observed | No | 1 |
| P57 | F | 33 | Acute-onset paroxysmal behavioral abnormalities for 1 day | Symptomatic epilepsy | 0 | Softening lesion in the right frontal lobe | No | 0 |
| P132 | F | 32 | Episodes of dizziness for 6 months, recurrent seizures for 2+ months | Seizure-like attacks | 0 | No significant abnormalities observed | No | 1 |
| P62 | F | 51 | Disordered speech and behavior after fever for 20+ days | Encephalitis | 2×10^6/L | 1. Mild small ischemic lesions in the left parietal lobe;  2. Mild brain atrophy | No | 1 |
| P64 | M | 25 | Acute-onset behavioral abnormalities for 2 days | Encephalitis | 1×10^6/L | No significant abnormalities observed | No | 1 |
| P66 | F | 46 | Recurrent fever and cough for 3 weeks, incoherent speech for more than 1 day | Delirium | 0 | Small spot lesions in bilateral frontal lobe white matter, suggestive of demyelination | No | 1 |
| P70 | M | 55 | Recurrent fever for 1 week, behavioral abnormalities for 2 days | 1. Delirium;  2. Lacunar infarction;  3. Nodular goiter | 5×10^6/L | No significant abnormalities observed | No | 1 |
| P76 | M | 18 | Psychiatric abnormalities with episodic facial twitching for 1+ year | Seizure-like attacks | 5×10^6/L | Small spot lesions in the right parietal lobe white matter, suggestive of demyelination | No | 2 |
| P77 | F | 29 | Acute-onset fever with behavioral abnormalities for 1 day | 1. Epilepsy;  2. Urinary tract infection | 6×10^6/L | No significant abnormalities observed | No | 1 |
| P123 | M | 56 | Acute-onset fever and cough for 12 days, behavioral abnormalities for 10 days | 1. Herpes simplex viral encephalitis;  2. Symptomatic focal epilepsy | 169×10^6/L | MRI: Abnormal signals in the left temporal hook, insula, and hippocampal head, suggesting encephalitis | No | 2 |
| P87 | F | 55 | Acute-onset behavioral abnormalities for 3 months | Neurosyphilis | 10×10^6/L | No significant abnormalities observed | No | 2 |
| P80 | M | 53 | Acute-onset memory decline with aphasia for 1 week | Neurosyphilis | 6×10^6/L | 1. Extensive abnormal signals in the left temporal lobe, possibly indicating inflammation; 2. Brain atrophy (including the hippocampus) | No | 2 |
| P86 | F | 63 | Recurrent dizziness and falls for 2 years, memory decline with behavioral abnormalities for 1 year | Neurosyphilis | 2×10^6/L | 1. Multiple ischemic lesions in the bilateral frontal and adjacent temporal lobes;  2. Possible degeneration of hippocampus and entorhinal cortex in the bilateral temporal lobes | No | 2 |
| P82 | F | 56 | Memory decline with behavioral abnormalities for 1 month | Neurosyphilis | 8×10^6/L | 1. Multiple ischemic lesions in the bilateral temporal and adjacent brain regions;  2. Mild hippocampal atrophy | No | 2 |
| P106 | F | 60 | Memory decline for 1 year, worsening for 3+ months | Neurosyphilis | 5×10^6/L | Diffuse white matter abnormalities beneath the bilateral frontotemporal-parietal cortices | No | 2 |
| P107 | M | 52 | Memory decline with behavioral abnormalities for 1+ month | Neurosyphilis | 0 | Slight ischemic lesions in bilateral frontal lobes | No | 2 |
| P142 | F | 14 | Depressed mood with delayed reactions for 3+ months | Encephalitis | 5×10^6/L | No significant abnormalities observed | No | 1 |
| P138 | F | 16 | Acute-onset delirium for 3 days | Delirium | 3×10^6/L | Small demyelinating lesions in bilateral frontal lobes | No | 0 |
| P139 | M | 28 | Acute-onset emotional instability and behavioral abnormalities for 5 days | Subclinical hyperthyroidism, psychiatric disorder caused by physical illness | 2×10^6/L | White matter lesions in bilateral frontal lobes, suggesting demyelination | No | 1 |
| P140 | F | 13 | Sudden-onset disorganized speech for 3 days | Acute stress disorder, delirium | 2×10^6/L | No significant abnormalities observed | No | 1 |
| P141 | M | 61 | Recurrent episodes of altered consciousness for 1+ year, recurrent episodes for 1 week | Seizure-like attacks | 1×10^6/L | 1. Possible old cerebellar hemisphere infarctions on the left side, possible subacute infarction cavity on the right side; 2. Brain atrophy | No | 1 |

**Supplementary Table 6** TBA showed a suspected AE patient with positive nuclear antibodies

| **Patient** | **Sex** | **Age (y)** | **Main Symptoms** | **Diagnosis** | **CSF (WBC)** | **MRI** | **Immunotherapy** | **Prognosis (mRS)** |
| --- | --- | --- | --- | --- | --- | --- | --- | --- |
| P61 | F | 68 | Acute memory decline for 6 days | Encephalitis | 6×10^6/L | Multiple abnormal signals in bilateral medial temporal lobes, hippocampus, and right insular lobe | Yes | 1 |
| P135 | M | 28 | Acute headache with behavioral and speech disturbances for 2 days | Intracranial venous sinus thrombosis | 5×10^6/L | Possible thrombosis in the right transverse and sigmoid sinuses | No | 0 |
| P90 | M | 54 | Excitement and excessive speech following a fall-related injury for more than a month | Neurosyphilis | 2×10^6/L | Multiple ischemic lesions in the bilateral basal ganglia, corona radiata, insular lobes, and pons | No | 1 |
| P95 | M | 47 | Memory decline with behavioral abnormalities for 3 months | Neurosyphilis | 8×10^6/L | Multiple ischemic lesions in the right basal ganglia and bilateral frontotemporal white matter regions | No | 1 |
| P119 | M | 13 | Incoherent speech for 4 days, recurrent seizures for 3 days | Epilepsy | 1×10^6/L | No significant abnormalities observed in brain and hippocampal MRI scans | No | 0 |
| P120 | M | 13 | Recurrent episodes of loss of consciousness with limb twitching for 2 years, worsened with irritability for 3 months | Epilepsy | 3×10^6/L | Possible left hippocampal sclerosis, ruled out focal cortical dysplasia in the left temporal lobe | No | 1 |
| P121 | M | 59 | Recurrent psychiatric symptoms for over 20 years, recent relapse with seizure-like episodes for 4 days | Herpes simplex virus encephalitis | 7×10^6/L | 1) Multiple ischemic lesions in the bilateral frontal, parietal lobes, and periventricular regions.  2) Mild cerebral white matter rarefaction.  3) Cerebral atrophy. | No | 1 |
| P124 | F | 65 | Memory decline for over 3 years, worsened with speech and behavioral disturbances for over 2 weeks | Encephalitis? | 19×10^6/L | Cerebral atrophy | No | 2 |
| P125 | F | 13 | Emotional instability for over a year, refusal to eat for 2 weeks | Encephalitis (Rickettsial infection); Atypical neurogenic anorexia | 2×10^6/L | Lesion in the pressure point of the corpus callosum, reversible corpus callosum syndrome is considered | No | 1 |
| P127 | F | 20 | Acute onset of altered behavior following fever for 5 months | Encephalitis? | 2×10^6/L | No significant abnormalities observed in MRI scans | No | 0 |
| P128 | M | 15 | Behavioral disturbances and self-laughter for over a month | Encephalitis? | 0×10^6/L | Leukoaraiosis in the periventricular regions of both lateral ventricles | No | 1 |
| P129 | F | 67 | Behavioral disturbances and depressed mood for over 20 days | 1. Encephalitis,  2. Bacterial pneumonia  3. Pulmonary nodules,  4. Type 2 diabetes | 5×10^6/L | 1. Bilateral periventricular ischemic changes.  2. Mild cerebral atrophy | No | 1 |
| P130 | M | 33 | Acute onset of behavioral disturbances for 3 days | 1. Organic mental disorder, 2. Connective tissue disease (SLE?) | 1×10^6/L | No significant abnormalities observed on brain MRI scans. MRV showed narrow lumens in the left transverse and sigmoid sinuses with partial unclear display | No | 1 |
| P134 | F | 23 | Low mood for 3 months, worsened with speech and behavioral abnormalities for 2 weeks | 1.Encephalitis,  2. Nodular thyroid disease | 5×10^6/L | Patchy abnormal signals below the left frontal cortex - left corona radiata | No | 1 |
| P99 | M | 58 | Behavioral disturbances and memory decline for 1 month | Neurosyphilis | 20×10^6/L | 1) Abnormal white matter signals beneath bilateral cerebral cortices, mainly in the temporal lobes, suggestive of encephalitis | No | 1 |
| P111 | M | 59 | Acute onset of behavioral disturbances for half a month | Neurosyphilis | 55×10^6/L | 1. Formation of small cavities in the left cerebellar hemisphere;  2. Cerebral white matter degeneration;  3. Mild diffuse cerebral atrophy | No | 1 |
| P118 | M | 68 | Memory decline for 3 months | Neurosyphilis | 2×10^6/L | 1) Multiple abnormal signals in bilateral frontal, parietal lobes, insular lobes, possibly ischemic lesions?  2) Cerebral white matter rarefaction, cerebral atrophy.  3)Hydrocephalus | No | 1 |
| P138 | F | 64 | Blurred vision, diplopia, headache for 1 month, worsened for 3 days | Pyogenic meningitis | 2489×10^6/L | 1) Changes in the pituitary gland and sella turcica.  2) Mild cerebral white matter rarefaction.  3) Mild cerebral atrophy. | No | 1 |
| P137 | M | 40 | Recurrent headaches for over 2 months, altered consciousness for 14 days | Tuberculous meningitis | 20×10^6/L | 1) Considered tuberculous meningitis with hydrocephalus, enlargement of the supratentorial ventricular system.  2) Multiple ischemic lesions in the pons, basal ganglia, and right corona radiata.  3) Cerebral white matter rarefaction | No | 1 |
